# Proteomic Profiling as a Diagnostic Biomarker for Discriminating Between Bipolar and Unipolar Depression

**DOI:** 10.3389/fpsyt.2020.00189

**Published:** 2020-04-17

**Authors:** Sarah Kittel-Schneider, Tim Hahn, Frieder Haenisch, Rhiannon McNeill, Andreas Reif, Sabine Bahn

**Affiliations:** ^1^Department of Psychiatry, Psychotherapy and Psychosomatic Medicine, University Hospital, University of Würzburg, Würzburg, Germany; ^2^Department of Psychiatry, Psychosomatic Medicine and Psychotherapy, University Hospital, Goethe-University of Frankfurt, Frankfurt, Germany; ^3^Department of Psychiatry, Psychotherapy and Psychosomatic Medicine, University Hospital, University of Münster, Münster, Germany; ^4^Department of Chemical Engineering and Biotechnology, University of Cambridge, Cambridge, United Kingdom

**Keywords:** affective disorder, bipolar disorder, major depression (MD), major depressive disorder (MDD), proteome, biomarker, blood, machine learning

## Abstract

**Introduction:**

Affective disorders are a major global burden, with approximately 15% of people worldwide suffering from some form of affective disorder. In patients experiencing their first depressive episode, in most cases it cannot be distinguished whether this is due to bipolar disorder (BD) or major depressive disorder (MDD). Valid fluid biomarkers able to discriminate between the two disorders in a clinical setting are not yet available.

**Material and Methods:**

Seventy depressed patients suffering from BD (bipolar I and II subtypes) and 42 patients with major MDD were recruited and blood samples were taken for proteomic analyses after 8 h fasting. Proteomic profiles were analyzed using the Multiplex Immunoassay platform from Myriad Rules Based Medicine (Myriad RBM; Austin, Texas, USA). Human DiscoveryMAP^TM^ was used to measure the concentration of various proteins, peptides, and small molecules. A multivariate predictive model was consequently constructed to differentiate between BD and MDD.

**Results:**

Based on the various proteomic profiles, the algorithm could discriminate depressed BD patients from MDD patients with an accuracy of 67%.

**Discussion:**

The results of this preliminary study suggest that future discrimination between bipolar and unipolar depression in a single case could be possible, using predictive biomarker models based on blood proteomic profiling.

## Introduction

Depressive episodes affect up to 322 million people worldwide (Depression and Other Common Mental Disorders. Global Health Estimates. Geneva: World Health Organization 2017, https://apps.who.int/iris/bitstream/handle/10665/254610/WHO-MSD-MER-2017.2-eng.pdf). People suffering from a depressive episode can be suffering from either unipolar depression (major depressive disorder; MDD) or bipolar affective disorder (BD) as the underlying cause, depending on whether previous (hypo)-manic episodes have occurred. Unfortunately, this distinction can only be made after the first (hypo-)manic episode has presented. Therefore the most appropriate treatment for the underlying disorder may not initially be prescribed, especially as BD and MDD require fundamentally different pharmacological approaches; BD requires mood stabilizing medication, whereas MDD is treated with antidepressant monotherapy as a first-line treatment (1–3). Patients suffering from BD are often misdiagnosed as MDD and therefore adequate treatment can be delayed for up to several years (4, 5). Inadequate and delayed treatment increases the direct and indirect economic cost of BD, augments individual suffering, and impairs the overall prognosis (6). However, despite distinct treatment approaches, BD and MDD appear to share common molecular pathomechanisms. The gradient of MDD polygenic risk sore has been shown to slide across the mood disorder spectrum, demonstrating an inverse relationship to the mania polygenic risk score (7).

The development of fluid biomarkers that can discriminate between BD and MDD would be highly beneficial, but reliable biomarkers have so far remained elusive. Nonspecific findings have been obtained in many studies, which failed to detect disorder-specific alterations, instead identifying molecular mechanisms implicated in several different psychiatric conditions. For example, several studies have reported dysregulation of the nitrinergic system in BD, but also in ADHD and schizophrenia (8–11). Recent work has additionally suggested that nitric oxide may play a role in the pathophysiology of major depression (12). Another potential cross-disorder mechanism is a dysfunctional hypothalamic-pituitary-adrenal axis (HPA axis), which has been implicated in both BD and MDD (13–15). Moreover, inflammatory processes (including the glucocorticoid system) may also play a role in MDD and BD (16, 17).

Despite several shared neurobiological features of psychiatric disorders, combining different modalities or vast arrays of biomarkers (e.g. using proteomic profiling) has demonstrated potential for providing disorder-specific biomarkers. A previous own study defined a diagnostic panel consisting of 20 protein analytes suitable for the diagnosis of BD (18). Additionally, Chen et al. published a set of 20 differential urine metabolites that could discriminate between BD and MDD (19). Although these initial findings are encouraging, the use of univariate statistical inference does not provide sufficient information to determine discriminative power for individual patients, nor does it quantify generalisation to new data. These initial promising results therefore need to be replicated in additional samples, and more importantly tested for personalized predictive power, before a diagnostic biomarker panel can be used in clinical routine. In addition, modern machine learning approaches may considerably increase model performance by considering multivariate patterns in the data, thereby improving upon classic univariate approaches. In this study, we investigated whether a multivariate machine learning approach using data from multiplexed proteomic assays could accurately be used to discriminate between BD and MDD.

## Materials and Methods

### Study Participants

Bipolar and major depression patients were part of a naturalistic sample recruited from patients treated in our in- and outpatient clinics. The male and female participants were within the age range 18–78 years, had a body mass index (BMI) between 18 and 46 kg/m, and had a test negative for recreational drug screening at the time of sampling. Patients were diagnosed with BD or MDD by two trained psychiatrists (SKS, AR) according to criteria of the International Classification of Diseases–10 (ICD-10), while being treated as inpatients or outpatients at the Department of Psychiatry, Psychosomatic Medicine and Psychotherapy of the University Hospital of Würzburg. Diagnoses were confirmed by the Operational Criteria Checklist for Affective and Psychotic Illness (OPCRIT) (20). Severity of symptoms was assessed using the standard questionnaire-based rating scales Young Mania Rating Scale (YMRS) and Montgomery–Åsberg Depression Rating Scale (MADRS) (21, 22).

Both bipolar I and bipolar II disorder patients were recruited and were in depressed mood states at the time of sample collection. MDD patients also had an acute depressive episode at the time of sample collection. Exclusion criteria included a diagnosis of severe coronary heart disease or cardiac insufficiency (i.e. coronary stent, cardiac bypass surgery angina pectoris, and cardiac insufficiency NYHA>I), severe autoimmune disorders (Hashimoto’s thyroiditis excluded), acute or chronic infections, treatment with immunosuppressive/immune-modulating drugs or antibiotics, other severe neuropsychiatric disorders, chronic terminal diseases affecting the brain (such as cancer or hepatic/renal insufficiency), and alcohol or drug addiction (self-reported or taken from hospital discharge letters/general practitioner’s letters). Patients were fasting for at least 8 h prior to blood sample collection. For more demographic details as well as somatic disorders and medication taken at sampling point see **Table 1** and **Supplemental Table 2**.

**Table 1:** Demographic data.

Depressive episode	n=70	n=42
	Bipolar Disorder	Major Depression
**BD I/BD II**	30/40	N/A
**Age (years, mean +/− SD)**	43.47 +/− 11.69	44.28 +/− 14.93
**Gender (female/male)**	44/28	25/16
**BMI**	27.50 +/− 5.72	27.78 +/− 6.27
**Disease duration (years, mean +/−SD)**	15.10 +/−11.32	N/A
**MADRS sum score (mean +/−SD)**	18.0 +/− 1.96	18.47 +/− 8.32
**YMRS sum score (mean +/−SD)**	2.0 +/− 0.22	N/A
**Medication**		
Lithium	7	3
Valproic acid	5	0
Other anticonvulsants	1	0
Antipsychotics	17	9
Lithium + Valproic acid	4	0
Valproic acid + antipsychotics	7	0
Other anticonvulsants + antipsychotics	4	0
Lithium + anticonvulsants + antipsychotics	4	0
Lithium + antipsychotics	21	1
Antidepressants only	2	28

BD I, bipolar disorder type 1; BD II, bipolar disorder type II; BMI, body mass index; MADRS, Montgomery-Åsberg Depression Scale; YMR, Young Mania Rating Scale; N/A, not available.

Only study participants who gave written informed consent were enrolled in the study, which complied with the latest Declaration of Helsinki, and was approved by the Ethics Committee of the University of Würzburg.

### Sample Collection

Patients were recruited over a total time period of 4 years (2009–2013), and therefore proteomic profiles were analyzed in four batches. Proteomic analyses were completed in 2010, 2011, and 2013. The maximum storage time for each sample in −80°C prior to analysis was 2 years.

### Sample Preparation

Blood samples were taken on the day of clinical assessment (± 24 h). Blood was obtained from the participants by venous puncture in the morning after fasting for 10–13 h (between 7 to 9 am). Serum was collected from fasting patients using Vacutainer (Becton-Dickinson, Franklin Lakes, NJ, USA). Blood clotting time was 2 h at room temperature prior to centrifugation for 15 min at 1.100 *x* g. Samples were stored in low binding Eppendorf reaction tubes (Hamburg, Germany) at −80°C. Sample shipment took place on dry ice.

### Multiplex Immunoassay Analysis

Serum from all participants was profiled using the multiplex immunoassay platform at Myriad Rules Based Medicine (Myriad RBM; Austin, Texas, USA), which has been previously described in detail (23). The Human DiscoveryMAP^TM^ was used to measure the plasma concentrations of different proteins, peptides and small molecules (collectively referred to as “analytes”), in a Clinical Laboratory Improved Amendments certified lab. The total number of analytes measured differed between batches, depending on when the study samples were profiled (total range: 190 to 257 analytes). The analytes measured are reported in **Supplemental Table 1** and the concentration of all the analytes for all participants are reported in **Supplemental Table 3** and **Supplemental Table 4**. The raw data of the four different multiplex assays were normalised for batch effects to reduce variability.

### Statistical Analysis

Statistical analyses were performed in R (24). We pre-processed the analyte data by excluding analytes with greater than 20% missing values, and imputing missing data. Data points under the lowest limit of detection (LLD) were replaced by the minimum value above the LLD for the specific analyte, and values above the highest detectable limit were replaced with the maximum measured valued within the detectable range. In total, 1.1% of data points were imputed. We log_10_-transformed data to stabilize the variance. Additional analyses were performed using SPSS (v25, IBM®). The two analytes that appeared to play a significant role in discriminating between unipolar and bipolar depression were analyzed separately using ANCOVA.

### Machine Learning Algorithm

In order to discriminate between patients suffering from MDD and BD, we first scaled all features (i.e. analytes) to have zero mean and unit variance. Next, tree ensemble classification was performed using the scikit-learn implementation of the AdaBoost algorithm with default hyperparameters (25, 26). To facilitate training in this extensively imbalanced dataset, we additionally employed Random Oversampling to the training set.

To assess the generalizability of the classifier, we used 10-fold cross-validation. Tenfold cross-validation is the most common standard in the field which ensures low model bias (due to the fairly large training sample) and low variance (due to the reasonably sized test set). Finding a balance between training and test sample size in each iteration is important, particularly because of the fairly small sample size used (for an introduction to the issue of k-fold cross-validation in practice see Bengio et al. (27). In each fold, data from 90% of the sample is used to train the classifier. Categorization of the remaining 10%, which has so far not been seen by the algorithm, is subsequently calculated. This procedure is repeated 10 times, each time leaving out different, nonoverlapping 10% portions of the sample, yielding each subject’s categorization. To ensure unbiased test performance estimates in this imbalanced sample, accuracy was computed by calculating the mean of sensitivity and specificity, yielding “balanced accuracy.”

To establish whether the observed test accuracy estimate is statistically significant, we ran the entire pipeline 1,000 times with randomly permuted labels and counted the number of permutations which achieved higher accuracy than the one observed with the true labels. The p-value was then calculated by dividing this number by 1,000. If none of the permutation accuracies exceeded accuracy obtained with the true labels, this is denoted as p<.001.

To quantify the contribution of each feature, we computed permutation importance scores, calculated as the mean decrease of test accuracy for all samples if a given feature is randomly shuffled 10 times. Generally, permutation importance as used here provides a measure of how much a feature contributed to classification performance while leaving all other features intact. All analyses were performed using the PHOTON framework (www.photon-ai.com).

## Results

**Figure 1:**
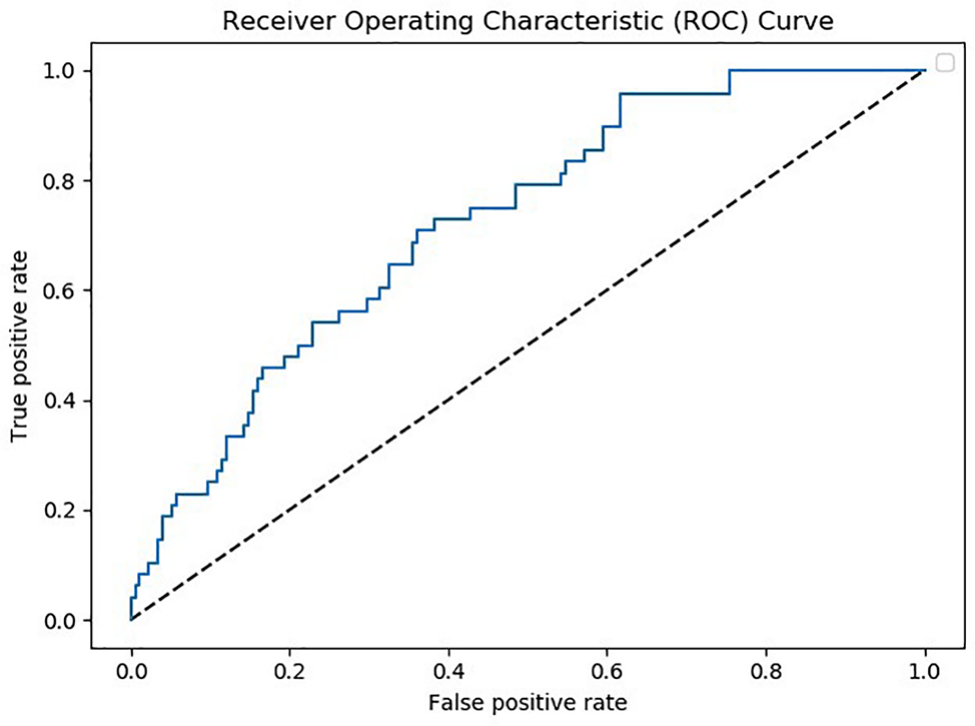
Receiver operating characteristic (ROC) curve depicting true positive versus false positive rates for the major depressive disorder (MDD) vs. bipolar disorder (BD) classification. Note that MDD was denoted as the positive class.

Patients with MDD and BD did not differ in basic demographic variables (see **Table 1**). However, medication significantly differed, as the majority of BD patients were taking mood stabilizers and the majority of MD patients were taking antidepressants (**Table 1**). Using data from 105 analytes, which was the lowest common denominator of the four batches, a multivariate predictive model was constructed to discriminate between MDD and BD (combined BD I and BD II disorders). The algorithm could discriminate between these two groups on the basis of the proteomic profile with an accuracy of 67% (p>.001 with 1,000 permutations) (see **Figure 1**). The analytes which the algorithm used for discrimination and prediction are displayed in **Supplemental Tables 1**, **3**, and **4**. The two analytes Platelet-Derived Growth Factor BB (PDGF-BB) and Thrombospondin-1 (TSP-1) were identified as particularly important for discriminating between BD and MDD in our sample. A subanalysis examining only young BD patients (<35 years) did not increase the accuracy of discrimination (data not shown). Additionally, it was observed that including a set of covariates (such as symptom severity and BD subtype) did not lead to improved accuracy (data not shown). To assess the potential influence of medication, we performed a covariate analysis using PDGF-BB and TSP-1 and included diagnosis (bipolar depression vs. MDD), medication, age, gender and BMI as covariates. No significant differences were found (p=0.19, p=0.47 respectively; see **Table 2**).

**Table 2:** TSP-1 and PDGF-BB levels.

	Disorder	N	Mean (µg/L)	Sth. Deviation +/−	ANCOVA, p
PDGF-BB	Bipolar Disorder	70	15,811.67	5,828.79	
	Major depression	42	16,641.46	6,515.39	0.19
TSP-1	Bipolar Disorder	70	16,498.75	8,180.89	
	Major depression	42	17,424.39	4,829.24	0.47

PDGF-BB, platelet-derived growth factor BB; TSP-1, thrombospondin-1; ANCOVA, analysis were calculated including diagnosis, age, gender, and medication as variables.

## Discussion

Recent studies found that ~10% of patients suffering from a depressive episode subsequently develop BD (4). To date, there are no tests in clinical routine to determine the risk of developing BD in patients experiencing their first depressive episode. However, it would be of great clinical significance to be able to accurately predict the underlying disorder, as pharmacological treatment differs considerably between MDD and BD. In this study, we have demonstrated that a machine-learning algorithm was able to individually discriminate BD (acutely depressed) from MDD (acutely depressed) patients with a moderately good accuracy of 67%, based on their proteomic profile. Based on our data, PDGF-BB and TSP-1 appeared to play a prominent role in this discrimination. Along with other serum analytes, PDGF-BB has previously been reported as associated with lower fractional anisotropy, higher mean diffusivity, and higher radial diffusivity in several brain regions in a sample of depressed BD patients (28). Furthermore, PDGF-BB was found to be increased in BD patients suffering a depressive episode after treatment with a combination of sleep deprivation, lithium and bright light therapy (29). PDGF-BB was also found to have low intra-individual variability when measured with different methods and in serum and plasma, suggesting that this marker may be technically reliable (30). Physiologically, PDGF receptors have been reported to play a role in glutamatergic signaling (31), which is thought to be dysregulated in subtypes of affective patients (32).

TSP-1 may be involved in synaptogenesis (33). Electroconvulsive therapy (ECT) is used to treat therapy-resistant MDD and BD depression, and was found to increase TSP-1 mRNA and protein expression in a rat model. However, chronic antidepressant treatment in this animal model appeared to have no effect on TSP-1 (34). Preclinical data has additionally suggested that TSP-1 may play a role in bidirectional neuron-astrocyte communication, dysregulation of which could be a pathomechanism for the development of mental illnesses (35). There are also several in vitro studies demonstrating that the mood stabilizer valproate can induce TSP-1 protein expression and thrombospondin-1 (*THBS-1*) gene expression in different cell and animal models (36–38). This is in contrast to our results, which showed no difference in TSP-1 expression between patients treated with valproate and patients treated with the other mood stabilizers and antidepressants. However, valproate may only exert its main effect on TSP-1 expression in the central nervous system, with its effects not detectable in the periphery. With regards to human in vivo data, a recent study reported decreased TSP-1 serum levels in female patients with MDD compared to healthy controls and male MDD patients. However, ECT treatment did not influence TSP-1 levels, leading the authors to conclude that serum TSP-1 may be a state marker of female MDD rather than a trait marker (39). Nonetheless, a technical issue in both our study and the one performed by Okada-Tsuchioka et al. is that TSP-1 concentration was measured in serum and not plasma. As thrombocytes release TSP-1 in high concentrations, the TSP-1 generated by other cells may be masked (40). Future studies should therefore measure plasma TSP-1.

Several studies have previously attempted to discriminate between MDD and BD patients using fluid biomarkers. A recent study from Chen and colleagues analyzed urinary metabolic phenotypes and demonstrated that a panel of six urinary metabolites could potentially be used to discriminate between the two disorders (19). In a study comparing cytokine concentrations between remitted BD and MDD patients and healthy controls, higher concentrations of soluble Interleukin-6 receptor (sIL-6R), C-reactive protein (CRP), soluble Tumor-Necrosis-Factor-receptor-1 (sTNF-R) and Monocyte-chemoattractant-protein -1 (MCP-1) were shown in BD compared to MDD (41). Frye et al. used a similar approach to our current and previous studies but with a smaller sample size of MDD patients, BD patients and healthy controls, and observed differences in several serum proteins between groups. To date, the best diagnostic accuracy (>0.8) for discriminating between BD I patients and healthy controls was shown by growth-differentiation factor 15 (GDF-15), retinol-binding protein (RBP-4) and transthyretin (TTR). However, in the same study no marker could be identified that accurately discriminated between BD and MDD, which would be the most clinically relevant diagnostic biomarker (42). GDF-15 and RBP-4 were not included as analytes in our studies and therefore no data comparisons can be made. However, Frye and colleagues also found six proteins to be significantly increased in depressed BD and MDD patients, and these were found to differ from those of our own previous multi-center study with the exception of MMP-7 (18). PDGF-BB and TSP-1 were also not found to be significant markers in our previous study, although our previous samples were derived from BD patients in all episodes (including euthymic), currently depressed and euthymic MDD patients, which could explain the differences in results. Whereas in this smaller sample we only compared acutely depressed bipolar patients vs. current major depression. This inconsistency could be therefore due to the differences in the samples which were investigated with respect to current episode and subtypes as well as to different proteins included in the analysis. Our current study additionally improves upon previous approaches, as it is the first to move beyond group-statistical inference to provide single-subject predictions. As individualized prediction is a key requirement for clinical application, our results support the clinical utility of multivariate predictive analytics approaches in the field.

A current limitation of using fluid peripheral biomarkers is that concentrations measured in the periphery do not necessarily reflect pathophysiological processes in the central nervous system. However, our primary aim is to develop a biomarker that can discriminate between disorders, and not identify underlying disease pathomechanisms. We therefore believe that the most important factor is whether biomarker expression varies sufficiently between individuals to allow for discrimination between different disorders, and not whether the biomarker is in directly involved in disease aetiology. However, for most single metabolites, the differences between groups are statistically significant but not great enough for single prediction [for examples, see (9, 39, 43, 44)]. We therefore suggest that currently the most promising approach for individual prediction is to measure several analytes simultaneously in the form of a biomarker panel, with additional machine learning, rather than measuring only a few select proteins.

Machine-learning algorithms in the development of diagnostic biomarkers have so far mainly been used in neuroimaging studies. There are several preliminary studies demonstrating the potential of this approach, for applications such as identifying individuals at high-risk of BD (45) and for defining subphenotypes of BD (46). However, machine learning may impair the algorithm’s ability to derive a high performing model, and preclude the use of more sophisticated approaches, potentially rendering our results an artificially low estimate of the true accuracy. Despite these limitations, the sample size used for evaluation in machine learning entails fairly small test sets, potentially increasing variance of performance estimates (although we employed cross-validation).

To conclude, the initial results obtained from our study are promising. However, larger samples of patients are needed to replicate the results, thereby supporting the development of diagnostic biomarkers which can be used in clinical routine. We are aware that in this hypothesis generating study we could only examine a discovery sample. The necessary next step is to validate our findings in a second, independent dataset which is however not readily available. We are currently reaching out to conduct according replication studies which will finally be the touchstone whether or not the pilot data presented here holds true or not.

## Limitation

The results of our study have several limitations. First, in the subanalysis, sample sizes were small. Second, as all patients were medicated an influence of mood stabilizing medication and antidepressants on serum proteins cannot be excluded. Further studies using increased sample sizes and including drug-naïve BD and MDD patients should be performed to overcome this methodological weakness. However, studies on drug-naïve patients are difficult to conduct due to ethical issues.

## Data Availability Statement

The original contributions presented in the study are included in the article/**Supplementary Material**, further inquiries can be directed to the corresponding author(s).

## Ethics Statement

The studies involving human participants were reviewed and approved by the Ethics Committee of the University of Würzburg. The patients/participants provided their written informed consent to participate in this study.

## Author Contributions

SK-S and AR recruited the patients and collected the sample. SK-S wrote the paper draft and did parts of the analysis. FH and SB conducted the multiplex proteomic analysis. FH did the preanalysis of the data and added to the manuscript draft. TH conducted the machine learning analysis and added to the manuscript draft. SB, AR, and RM took part in writing the final manuscript. RM revised the language of the final revised manuscript.

## Funding

This study has been supported by the BMBF (BipoLife, TPP1 subproject to AR) and TH was supported by the German Research Foundation (DFG grants HA7070/2-2, HA7070/3, and HA7070/4) as well as LOEWE grant no 21000831. This publication was funded by the Goethe-University of Frankfurt.

## Conflict of Interest

SK-S has received speaker’s and author’s honoraria from Medice and Takeda. AR has received speaker fees and honoraria (publications, advisory boards) from Medice, Shire/Takeda, Servier, neuraxpharm, Janssen and SAGE.

The remaining authors declare that the research was conducted in the absence of any commercial or financial relationships that could be construed as a potential conflict of interest.
