# Supplementary material for: Proteomic Profiling as a Diagnostic Biomarker for Discriminating Between Bipolar and Unipolar Depression
Source: Front Psychiatry. 2020 Apr 17;11:189. doi: 10.3389/fpsyt.2020.00189 (PMC7184109; doi:10.3389/fpsyt.2020.00189)
Supplement: Supplementary file 1 [file Table_1.docx]

**Supplementary material**

Supplemental Table 1: List of commonly measured analytes after quality control.

|  | **Analyte** |  |  | **Analyte (continued)** |
| --- | --- | --- | --- | --- |
| [1] | Adiponectin |  | [54] | Interleukin-18 (IL-18) |
| [2] | Alpha-1-Antichymotrypsin (AACT) |  | [55] | Leptin |
| [3] | Alpha-1-Antitrypsin (AAT) |  | [56] | Luteinizing Hormone (LH) |
| [4] | Alpha-1-Microglobulin (A1Micro) |  | [57] | Macrophage-Derived Chemokine (MDC) |
| [5] | Alpha-2-Macroglobulin (A2Macro) |  | [58] | Macrophage Inflammatory Protein-1 beta (MIP-1 beta) |
| [6] | Angiopoietin-2 (ANG-2) |  | [59] | Macrophage Migration Inhibitory Factor (MIF) |
| [7] | Angiotensin-Converting Enzyme (ACE) |  | [60] | Matrix Metalloproteinase-1 (MMP-1) |
| [8] | Apolipoprotein(a) (Lp(a)) |  | [61] | Matrix Metalloproteinase-3 (MMP-3) |
| [9] | Apolipoprotein A-I (Apo A-I) |  | [62] | Matrix Metalloproteinase-7 (MMP-7) |
| [10] | Apolipoprotein A-II (Apo A-II) |  | [63] | Matrix Metalloproteinase-9, total (MMP-9, total) |
| [11] | Apolipoprotein A-IV (Apo A-IV) |  | [64] | Matrix Metalloproteinase-10 (MMP-10) |
| [12] | Apolipoprotein B (Apo B) |  | [65] | Monocyte Chemotactic Protein 1 (MCP-1) |
| [13] | Apolipoprotein C-I (Apo C-I) |  | [66] | Monocyte Chemotactic Protein 2 (MCP-2) |
| [14] | Apolipoprotein C-III (Apo C-III) |  | [67] | Monocyte Chemotactic Protein 4 (MCP-4) |
| [15] | Apolipoprotein D (Apo D) |  | [68] | Monokine Induced by Gamma Interferon (MIG) |
| [16] | Apolipoprotein E (Apo E) |  | [69] | Myeloid Progenitor Inhibitory Factor 1 (MPIF-1) |
| [17] | Apolipoprotein H (Apo H) |  | [70] | Myeloperoxidase (MPO) |
| [18] | AXL Receptor Tyrosine Kinase (AXL) |  | [71] | Myoglobin |
| [19] | Beta-2-Microglobulin (B2M) |  | [72] | Neutrophil Gelatinase-Associated Lipocalin (NGAL) |
| [20] | Brain-Derived Neurotrophic Factor (BDNF) |  | [73] | Osteopontin |
| [21] | C-Peptide |  | [74] | Pancreatic Polypeptide (PPP) |
| [22] | C-Reactive Protein (CRP) |  | [75] | Placenta Growth Factor (PLGF) |
| [23] | CD 40 antigen (CD40) |  | [76] | Plasminogen Activator Inhibitor 1 (PAI-1) |
| [24] | CD40 Ligand (CD40-L) |  | [77] | Platelet-Derived Growth Factor BB (PDGF-BB) |
| [25] | CD5 Antigen-like (CD5L) |  | [78] | Progesterone |
| [26] | Chemokine CC-4 (HCC-4) |  | [79] | Prolactin (PRL) |
| [27] | Chromogranin-A (CgA) |  | [80] | Pulmonary and Activation-Regulated Chemokine (PARC) |
| [28] | Clusterin (CLU) |  | [81] | Receptor for advanced glycosylation end products (RAGE) |
| [29] | Complement C3 (C3) |  | [82] | Resistin |
| [30] | Cortisol (Cortisol) |  | [83] | Serotransferrin (Transferrin) |
| [31] | Creatine Kinase-MB (CK-MB) |  | [84] | Serum Amyloid P-Component (SAP) |
| [32] | Cystatin-C |  | [85] | Sex Hormone-Binding Globulin (SHBG) |
| [33] | E-Selectin |  | [86] | Sortilin |
| [34] | EN-RAGE |  | [87] | Stem Cell Factor (SCF) |
| [35] | Eotaxin-1 |  | [88] | Superoxide Dismutase 1, soluble (SOD-1) |
| [36] | Epidermal Growth Factor (EGF) |  | [89] | T-Cell-Specific Protein RANTES (RANTES) |
| [37] | Epithelial-Derived Neutrophil-Activating Protein 78 (ENA-78) |  | [90] | Tamm-Horsfall Urinary Glycoprotein (THP) |
| [38] | Factor VII |  | [91] | Tenascin-C (TN-C) |
| [39] | FASLG Receptor (FAS) |  | [92] | Testosterone, Total |
| [40] | Ferritin (FRTN) |  | [93] | Thrombospondin-1 |
| [41] | Fetuin-A |  | [94] | Thyroid-Stimulating Hormone (TSH) |
| [42] | Follicle-Stimulating Hormone (FSH) |  | [95] | Thyroxine-Binding Globulin (TBG) |
| [43] | Haptoglobin |  | [96] | Tissue Inhibitor of Metalloproteinases 1 (TIMP-1) |
| [44] | Hepatocyte Growth Factor (HGF) |  | [97] | TNF-Related Apoptosis-Inducing Ligand Receptor 3 (TRAIL-R3) |
| [45] | Immunoglobulin A (IgA) |  | [98] | Transthyretin (TTR) |
| [46] | Immunoglobulin M (IgM) |  | [99] | Trefoil Factor 3 (TFF3) |
| [47] | Insulin |  | [100] | Tumor necrosis factor receptor 2 (TNFR2) |
| [48] | Insulin-like Growth Factor-Binding Protein 2 (IGFBP-2) |  | [101] | Vascular Cell Adhesion Molecule-1 (VCAM-1) |
| [49] | Intercellular Adhesion Molecule 1 (ICAM-1) |  | [102] | Vascular Endothelial Growth Factor (VEGF) |
| [50] | Interferon gamma Induced Protein 10 (IP-10) |  | [103] | Vitamin K-Dependent Protein S (VKDPS) |
| [51] | Interleukin-6 receptor (IL-6r) |  | [104] | Vitronectin |
| [52] | Interleukin-8 (IL-8) |  | [105] | von Willebrand Factor (vWF) |
| [53] | Interleukin-16 (IL-16) |  |  |  |
