# Supplementary material for: Proteomic Profiling as a Diagnostic Biomarker for Discriminating Between Bipolar and Unipolar Depression
Source: Front Psychiatry. 2020 Apr 17;11:189. doi: 10.3389/fpsyt.2020.00189 (PMC7184109; doi:10.3389/fpsyt.2020.00189)
Supplement: Supplementary file 2 [file Table_2.docx]

Supplementary Table 2: Somatic disorders and medication

| **Psychiatric diagnosis** | **Somatic disorder/Somatic history** | **Medication** |
| --- | --- | --- |
| Bipolar I | Begnin prostate hyperplasia | Allopurinonel 300mg, Flupirtin 100-0-100-0 mg, Bisoprolol 2,5 mg, Torasemid 20mg, Tamsulosin 0,4 mg |
| Bipolar I | Wasp allergy | none |
| Bipolar I | art. Hypertension, diabetes mellitus type II | L-Thyroxinµg, Enalapril 20 mg, Sitagliptin/Metformin 50/1000 mg 1-0-1-0 Tbl., Lactulose (z.B. Bifiteral) 30-30-30-0 ml |
| Bipolar I | Psoriasis, Tinnitus | none |
| Bipolar I | art. Hypertension, diabetes mellitus type II | Belok 23,75, Adalat |
| Bipolar I | Discus prolaps | Laxofalk |
| Bipolar I | Hysterectomy, Appendectomy | none |
| Bipolar I | Art. Hypertension, Cholezystectomy und Appendectomy | Pipamperon 40mg, Amlodipin 2,5mg, Metoprolol 95mg, Ramipril 15mg, ASS 100mg |
| Bipolar I | Commotio cerebri | none |
| Bipolar I | none | none |
| Bipolar I | none | none |
| Bipolar I | Acne | none |
| Bipolar I | Tonsillectomie, Neurodermatitis | none |
| Bipolar I | Asthma bronchiale, Tinnitus, Art. Hypertension | Bisoprolol, Symbicort |
| Bipolar I | nonene | Taxilan |
| Bipolar I | Preterm birth, Appendectomy | ACC |
| Bipolar I | allergies | none |
| Bipolar I | Appendectomy, Tonsillectomy | L-Thyroxin, Jodid |
| Bipolar I | nonene | Truxal, Magnesium, Lactulose |
| Bipolar I | allergies | none |
| Bipolar I | Struma, von-Willebrand-Jürgens-Syndrome, Tonsillectomy und Appendectomy | L-Thyroxin, Kaliumjodid |
| Bipolar I | art. Hyperonsion, Diabetes mellitus Typ II, Tinnitus aurium, Z. n. Appendectomy, Herniotomy | Bisoprolol, L-Thyroxin, Allopurinonel, Bezafibrat, Sitagliptin, Metformin, ASS, Pantoprazol |
| Bipolar I | Divertikulitis, Morbus Meulengracht, allergies | none |
| Bipolar I | none | L-Thyroxin |
| Bipolar I | thyroid hypofunction | L-Thyroxin |
| Bipolar I | art. Hypertension | Enalapril |
| Bipolar I | Tonsillectomy, allergy | none |
| Bipolar I | art. Hypertension, Appendectomy | Ramipril 5 mg, Metoprolol 95 mg |
| Bipolar I | obesity | Metoprolol |
| Bipolar I | Tonsillectomy und Appendectomy, Hypercholesterinemia, allergy | Simvastatin 10 mg, Pantoprazol, Laxofalk |
| Bipolar II | migraine, Appendectomy | Bisoprolol, Akineton |
| Bipolar II | benign prostate hyperplasia, allergy | Tamsulosin |
| Bipolar II | art. Hypertension, thyroid hypofunction, obesity | Torasemid, Irbesartan, L-Thyroxin, Bisoprolol |
| Bipolar II | Diabetes mellitus Type II, discus prolaps, Ulcus duodeni | Insulin, Voltaren, Pantozol |
| Bipolar II | Commotio cerebri | Zolpidem |
| Bipolar II | discus prolaps, reflux oesophagitis, hip and shoulder arthrosis | Pantoprazol 80 mg |
| Bipolar II | none | none |
| Bipolar II | Colitis ulcerosa, Hashimoto-Thyreoiditis | Prednisolon 5 mg, Mercaptopurin 50 µg, L-Thyroxin 50 µg, Vitamin D, Calcium. Pantoprazol 40 mg |
| Bipolar II | art. Hypertension, Dyslipidemia, discus prolaps, thyreoidectomy | Jodid, L-Thyroxin, Simvahexal, Aprovel |
| Bipolar II | art. Hypertension, chronic headaches | ASS 100mg, Torasemid 10m, Pantozol 40 mg, Enalapril 5 mg, HCT 12,5mg, Macrogol Btl. |
| Bipolar II | art. Hypertension, Appendectomy | Spirononelacton 100 mg, Bisoprolol 5 mg |
| Bipolar II | Appendectomy | Femigoa (Pille) |
| Bipolar II | art. Hypertension, benign prostate hyperplasia, Diabetes mellitus type II, hypercholesteremia , reflux oesophagitis, thyreoidectomy | Metoprolol 142,5 mg, L-Thyroxin 125µg, Cynt 40 mg, Tamsulosin 0,4 mg, HCT 12,5mg, Pantoprazol 40 mg, Aprovel 300 mg |
| Bipolar II | retinal detachment | Tamsulosin |
| Bipolar II | thyroid hypofunction | Thyranajod, Laxofalk |
| Bipolar II | knee surgery | Ergenyl Chronone |
| Bipolar II | allergies, Sjögren-Syndrome, hip arthrosis | Prednisolon, Vigantoletten, Jodid, Laxofalk, Pantoprazol |
| Bipolar II | Asthma, allergies, Polypectomy, Tinnitus | N/A |
| Bipolar II | none | none |
| Bipolar II | none | none |
| Bipolar II | thyroid hypofunction, Asthma, obesity | L-Thyroxin, Salbutamol Spray, Pregabalin |
| Bipolar II | allergic Asthma bronchiale, Appendectomy, mild Hämophilia A | none |
| Bipolar II | thyroid hypofunction | L-Thyroxin, Pantoprazol, Propranonelol |
| Bipolar II | Struma | L-Thyroxin 50µg |
| Bipolar II | none | none |
| Bipolar II | none | Medikinet, Valette (Pille), L-Thyroxin |
| Bipolar II | art. Hypertension, Diabetes mellitus Typ II, obesity | Fenonefibrat, Sitagliptin, Metformin, Lisinonepril, Bisoprolol |
| Bipolar II | Tonsillectomy | none |
| Bipolar II | art. Hypertension, SAE, Osteoporosis, migraine, Hashimoto-Thyreoiditis | L-Thyroxin 100µg, Ramipril 2,5 mg, Xipamin 10 mg, ASS 100 mg, Pantozol 40 mg, Vitamin D, Calcium, Magnesium, Bifiteral |
| Bipolar II | N/A | N/A |
| Bipolar II | Fibromyalgie, Gastric bybass | Tramal, Tolperison, Ramipril |
| Bipolar II | Diabetes mellitus Typ II, art. Hypertension, Z.n. Tonsillectomy, Z.n. Cholezystectomy, benign Prostatahyperplasia, discus prolaps | Glimepirid 3 mg, Metformin 3x850 mg, Metoprolol 95 mg, Aprovel 150 mg, Tamsulosin 0,4mg, Spasmex 2x15 mg, Seroquel 100 mg, L-Thyroxin 50µg |
| Bipolar II | chronic pain syndrome | Indometacin, Pantoprazol |
| Bipolar II | Psoriasis, discus prolaps | none |
| Bipolar II | art. Hypertension, allergy, dyslipidemia | Bisoprolol, HCT, Ramipril |
| Bipolar II | preterm birth, Tonsillectomy | Hormon-Spirale |
| Bipolar II | Hashimo-thyreoiditis, Endometriosis | L-Thyroxin |
| Bipolar II | Discus prolaps | none |
| Bipolar II | art. Hypertension, migraine, Struma, discus prolaps, Appendectomy | Topiramat, HCT, Sumatriptan |
| Bipolar II | allergies | none |
| Major Depression | allergies, Appendectomy, Tonsillectomy | Pantoprazol |
| Major Depression | Hypercholesterinemia; arterielle Hypertension | Pantoprazol, Metoprolol, Simvastatin, Carbamazepin |
| Major depression | art. Hypertension | Bisoprolol, Equilibrin |
| Major depression | Strumectomy | L-Thyroxin, Jodid |
| Major depression | None | Pantoprazol |
| Major depression | None | Magnesium |
| Major depression | Hypercholesterinemia | None |
| Major depression | N/A | N/A |
| Major depression | Art. Hypertension, COPD, Glaukoma, Mastectomy | N/A |
| Major depression | allergies, appenectomy | 40 mg Pantozol, Orthomol vital |
| Major depression | none | Pregabalin 225 mg, Zolpidem 10 mg |
| Major depression | thyroid hypofunction | L-Thyroxin 200 µg |
| Major depression | lactose intolerance | none |
| Major depression | art. Hypertension, Restless Legs, Strumectomy, allergic Asthma, allergies | Ropinirol, Ramipril, ACC, Viani und Berodual Spray |
| Major depression | hypercholesteremia , thyroid hypofunction, M. Ledderhose, reflux oesophagitis | Pregabalin, Esomeprazol, Atorvastatin, Jodthyrox |
| Major depression | hypercholesteremia , chronic Sinusitis | Simvastatin 20 mg, ACC long, Cefpocloxim |
| Major depression | allergy | Magnesium |
| Major depression | Art. Hypertension, thyreoidectomy | Propranonelol 30 mg, Amlodipin 10 mg, HCT 12,5 mg, Valsartan 160 mg, L-Thyroxin 100 µg, Macrogol, LaFamme |
| Major depression | history of tuberculosis, thyroid hypofunction | none |
| Major depression | Neurodermatitis, Asthma, allergies | none |
| Major depression | thyroid hypofunction, reflux oesophagitis | Pregabalin 300 mg, Pantoprazol 40 mg, Metoprolol 142,5 mg, Hydrochlorothiazid 12,5 mg, L-Thyroxin 50 µg |
| Major depression | Art. Hypertension, Diabetes mellitus Type II | Valsartan 80 mg, Metformin 850 mg |
| Major depression | Schlafapnoe, discud prolaps | Ramipril 5 mg, Ibuprofen 1600 mg, Pantoprazol 40 mg |
| Major depression | Restless Legs, hysterectomy, Adnexectomy, Endometriosis | Zolpidem 10mg, L-Dopa 100 mg, Vitamin B12 und Folsäure 3x5 mg und Neurotrat 3x100 mg |
| Major depression | Tinnitus, art. Hypertension, thyroid hypofunction, tension headaches, Appendectomy | Metoprolol 47,5 mg, Ramipril 5 mg, Jodod 200µg, Pantoprazol 40 mg, Mydocalm 2x50 mg |
| Major depression | metabolic syndrome, intermitt. art. Fibrillation , thyreoidectomy, art. Hypertension, Hypercholsteremia, obesity, allergy | Diovan 160mg, Amlodipin 10 mg, Bisoprolol 5 mg, L-Thyroxin 150µg, Marcumar |
| Major depression | TBI, thyroid hypofunction, Tonsillectomy, Appendectomy | L-Thyroxin 75 µg |
| Major depression | Neurodermatitis | none |
| Major depression | Art. Hypertension | Ramipril 5 mg |
| Major depression | TBI, Glaucoma, Appendectomy, allergies | Zolpidem 10 mg, Bisoprolol 5 mg |
| Major depression | Diabetes mellitus Type I, art. Hypertension, Z. n. Tonsillectomy | Enalapril 20 mg, Lercanidipin 10 mg, Insulin |
| Major depression | Diabetes mellitus type II, obesity per magna, art. Hypertension, Hyperunrikemia, allergies | Aldactone 100 mg, Allopurinonel 450 mg, Metoprolol 95 mg, Metformin 2000 mg, Ramipril 10 mg, Pantoprazol 40 mg, Dipiperon 40 mg, contraceptives |
| Major depression | Art. Hypertonsion, sleep apnoe, Appendectomy | Pregabalin 150 mg, Ambroxol 120 mg, Floxal |
| Major depression | Allergic Asthma, allergies, constipation | laxatives |
| Major depression | Asthma bronchiale | Contraceptives |
| Major depression | allergies | none |
| Major depression | none | none |
| Major depression | N/A | N/A |
| Major depression | allergies, allergic Asthma bronchiale | N/A |
| Major depression | Art. Hypertension, COPD | N/A |
| Major depression | none | none |
| Major depression | art. Hypertension | Ramipril |
